# Social Exclusion and Impulsive Buying among Chinese College Students: The Mediating Role of Self-Esteem and the Moderating Role of Risk Preference

**DOI:** 10.3390/ijerph182111027

**Published:** 2021-10-20

**Authors:** Haocheng Luo, Jiarong Chen, Shengnan Li, Yangang Nie, Guodong Wang

**Affiliations:** Research Center of Adolescent Psychology and Behavior, Department of Psychology, School of Education, Guangzhou University, Guangzhou 510006, China; luo_haocheng@yeah.net (H.L.); chenjiarong1021@yeah.net (J.C.); lishengnan010@gmail.com (S.L.); WGD20210424@163.com (G.W.)

**Keywords:** social exclusion, self-esteem, impulsive buying, risk preference, college student

## Abstract

With the development of science and technology, buying has become much easier. At the same time, however, impulsive buying has many negative consequences for college students, such as dissatisfaction and debt; the causes of impulsive buying should, therefore, be explored urgently. There are numerous empirical studies indicating that social exclusion may be a potential factor of impulsive buying, and the underlying mechanisms of this association remain unclear. In this study, we used the Social Exclusion Scale, Self-Esteem Scale, Risk Preference Scale, and Impulsive Buying Scale, as well as a cross-sectional design to investigate the roles of self-esteem and risk preference in the relationship between social exclusion and impulsive buying among 768 college students (387 were female, *M_age_* = 20.25 years). The results were as follows: (1) when controlling for gender, age, family monthly income, and monthly living expenses, social exclusion significantly and positively predicted impulsive buying; (2) self-esteem played a mediating role between social exclusion and impulsive buying; (3) risk preference moderated the relationship between the second half of the mediating path and the direct path. These results reveal the mechanism underlying impulsive buying in college students, that is, social exclusion will predict the decrease in college students’ self-esteem, and low self-esteem will further predict college students’ impulsive buying, which is a way for them to gain a sense of self-worth. Relatively low risk preference can well alleviate the negative impact of social exclusion and low self-esteem on impulsive buying. What is more, these results have implications for impulsive buying interventions and preventions. Schools should aim to create a good peer atmosphere by implementing certain rules that help to reduce social exclusion, and parents and education departments should cultivate students’ risk awareness to avoid risk behaviors in college students, such as impulsive buying behavior. This study fills the research gap regarding college students’ impulsive buying and explores its internal psychological mechanism.

## 1. Introduction

Shopping has become an indispensable part of college students’ lives. According to an official report, the proportion of Internet users who shop online has reached 79.1% in China [1]. Most of the college students are still in their late adolescence and they have gradually become increasingly targeted by marketers [2]. Moreover, with the rapid development of science and technology, such as online shopping and express service, shopping has become even more convenient. However, it has also caused some problems for college students, one of which is impulsive buying. In China, college students have more disposable money than junior high school students and senior high school students, but the correct concept of consumption has not been fully formed, so the possibility of impulsive buying is also higher. What is more, a large number of previous studies have shown that social exclusion will have a serious and far-reaching negative impact on college students, such as internet addiction [3] and depression [4]. Therefore, it is of great significance to explore the relationship between social exclusion and college students’ impulsive buying.

Impulsive buying is defined as sudden and unplanned buying behavior that is driven by a strong and persistent impulse, after which consumers experience a series of emotional, cognitive, and/or behavioral traits [5]. Consumers are now more inclined than ever to be utilitarian and engage in hedonic buying [6], and they enjoy the feeling of shopping more than buying what they really need [7]. While impulsive buying can bring immediate enjoyment and satisfaction [8], it is also closely related to some adverse consequences, such as low self-esteem, dissatisfaction [9], and debt [10]. The changes in an individual’s emotion and cognition following impulsive buying can lead to the subsequent recurrence of impulsive buying [5]. College students in late adolescence, who have not yet achieved mature thinking or economic independence, might engage in continuously excessive and uncontrollable buying if they have an improper shopping style, which could, in turn, cause more negative consequences, as mentioned. Although a large number of previous studies have discussed the negative consequences of impulsive buying on college students, its internal mechanism is not clear. Therefore, it is vital to explore the causes of impulsive buying among adolescences to intervene and prevent it.

## 2. Literature Review

### 2.1. Social Exclusion and College Students’ Impulsive Buying

Ecological systems theory regards development as a process of “individual–environment” interaction [11,12]. For adolescents, peers are the group environment with which they interact, and which might have a direct or indirect impact on their behavior and mental health, manifesting in antisocial behavior and depression symptoms [13]. For college students in late adolescence or just after adolescence, it may have a similar effect. Social exclusion as a kind of peer relationship deserves attention as a potential cause of college students’ negative behaviors or moods [14]. Social exclusion is a negative social phenomenon that manifests as exclusion, isolation, and rejection. Being excluded might stop individuals from developing relationships and pursuing a sense of belonging [15]. From the perspective of emotion and mood, previous studies have shown that individuals described experiencing pain when they were socially excluded, even though they were not physically injured [16]. College students might tend to distract and relieve themselves from this pain by engaging in other activities, such as Internet or alcohol use [17,18,19]. According to Twenge et al. [20], social exclusion makes individuals unconsciously choose out-of-control behaviors, such as high-risk and unhealthy behaviors.

As a high-risk behavior, impulsive buying is also highly likely to be predicted by social exclusion. Indeed, given that impulsive buying can make individuals feel immediately satisfied [8] and has the characteristics of high-risk behavior, which could lead to many negative consequences [10], it might be caused by social exclusion. Previous studies have also shown that customers can improve their negative mood by immediately buying products that bring satisfaction [21]. From a cognitive perspective, individuals may use cognitive resources to repair the negative effects of social exclusion, such as low self-esteem [22]. As a result, individuals might invest fewer cognitive resources into cognitive tasks, which affects their reasoning and decision-making abilities, and further increases impulsive buying. Furthermore, social exclusion can impair an individual’s self-regulation ability [23] and weaken their ability to inhibit impulsive behaviors [24], including impulsive buying. In addition, social exclusion could weaken intelligent thought [25]. In that case, individuals’ reasoning might be less rational, such that they are more easily controlled by their emotions and resulting in impulsive behaviors, such as impulsive buying [9]. Therefore, we proposed the following hypothesis:

**Hypothesis** **1** **(H1).**  *Social exclusion has a positive predictive impact on impulsive buying.*

### 2.2. Self-Esteem as a Mediator

Although social exclusion may be a direct predictor of impulsive buying, more research about the internal mechanisms, such as mediating influences, is needed to improve our understanding of impulsive buying and aid the development of effective interventions. Based on social exclusion theory and sociometer theory [14,26], individual’s interpersonal relationship will be reflected through self-esteem and individuals are eager to be accepted by peers to get a sense of belonging and security. A lack of peer group acceptance will lead to a series of negative consequences, such as anxiety, loneliness, depression, and low self-esteem [14]. 

Self-esteem is an attitude one has toward one’s self, and it is a mental representation of self-worth and self-acceptance [27]. Previous research has found that social exclusion induced by the Cyberball paradigm is also associated with decreased self-reported self-esteem, as well as reduced implicit self-esteem [28]. This shows that negative social feedback can affect an individual’s self-esteem [29]. Previous studies have also found that self-esteem is closely related to an individual’s interpersonal relationships [30], and that student–student relationships will directly affect individual self-esteem [31]. Therefore, it can be speculated that social exclusion might directly impact college students’ self-esteem. Moreover, low self-esteem might lead to self-doubt and a lack of self-confidence, such that college students might pay more attention to being accepted by society. To dissipate the negative emotions caused by low self-esteem, college students may gain self-worth through impulsive buying to compensate for a lack of social relationships [32]. Furthermore, Dittmar et al. [33] reported that buying products has become a way to obtain and express a sense of self-identity. College students who are excluded from society might buy impulsively to achieve a level of self-expression and form social ties through shopping [34]. Empirical research has also found that self-esteem mediates the relationship between mindfulness and impulsive buying tendencies [35]. Therefore, we proposed the following hypothesis:

**Hypothesis** **2** **(H2).**  *Self-esteem will mediate the relationship between social exclusion and impulsive buying.*

### 2.3. Risk Preference as a Moderator

Although social exclusion may significantly impact college students’ impulsive behavior, not all college students with high levels of social exclusion will develop impulsive behaviors. According to the ecological systems theory [11,12], individuals’ development stems from the interplay between the environment (such as their social environment) and their intrapersonal characteristics (such as risk preference). Risk preference is another potentially important factor that influences impulsive decision-making and risk decision-making, and may, thus, affect impulsive buying. Risk preference refers to a person’s preferred reaction in the face of risk choice and safety choice [36,37]. High risk preference is usually associated with non-adaptive and impulsive behaviors, including drinking, taking drugs, smoking, gambling, and engaging in unsafe sexual behaviors [38,39,40]. As an impulsive behavior that can bring immediate satisfaction [8], impulsive buying also has certain risks; individuals often need to spend more money to get temporary happiness, and this process may be affected by differences in individual risk preference. Farley [41] has divided consumers into risk-seeking and risk-averse consumers. Risk-averse consumers focus on minimizing risk; their actions lead to hesitation and consideration. In other words, they seek security and stability. On the contrary, risk-seeking consumers are willing to face risks [42]. College students with high risk preference may make more impulsive decisions, such as impulsive buying, and prioritize the short-term benefits of impulsive buying over the losses. Therefore, college students with high risk preference who are excluded by society may find it more difficult to inhibit impulsive buying. However, college students with low risk preference may engage less in impulsive buying and may pay more attention to the negative effects brought by impulsive buying, such as short-term economic debt [8]. Even if these individuals experience high levels of social exclusion, they will consider the negative consequences of impulsive buying so as to restrain this behavior. 

To a certain extent, low risk preference may, therefore, restrain impulsive buying. We hypothesized that low risk preference is a positive factor that influences impulsive behavior and can largely overcome the negative impact of social exclusion and a lack of self-esteem. Previous research has also shown that low risk preference is directly related to high self-control [43] and that high self-control can inhibit impulsive behavior [44]. Therefore, we proposed the following hypothesis:

**Hypothesis** **3** **(H3).**  *Risk preference will moderate the direct and indirect link between social exclusion and impulsive buying. Specifically, the indirect association and the direct association between social exclusion and impulsive buying via self-esteem will be stronger in college students with high risk preference and will be weaker in college students with low risk preference.*

**Figure 1:**
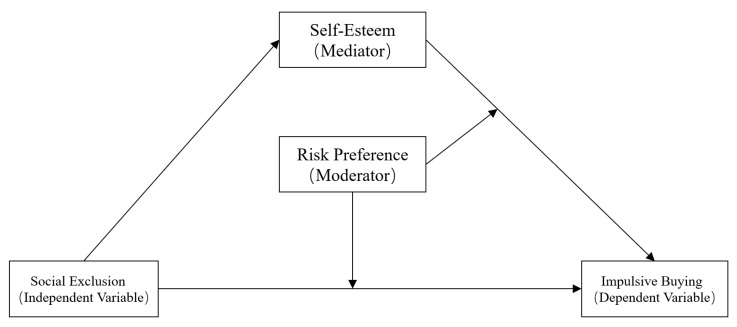
The proposed moderated mediation model.

In summary, this study proposed a moderated mediation model to explore the internal psychological mechanisms underlying the effect of social exclusion on impulse buying. The present results enhance our understanding of the mechanism underlying impulsive buying and provide a theoretical basis for the development of preventative measures and interventions for impulsive buying in college students. Figure 1 illustrates the proposed research model.

## 3. Methods

### 3.1. Participants

In this study, 811 college students from Guangzhou University, Guangdong University of Technology, Guangdong University of Finance and Economics, and other schools were surveyed using convenience sampling. The survey was conducted anonymously. After completing the questionnaire, respondents received a reward of CNY 1. The valid sample used in the analysis comprised 768 respondents (94.7% response rate; *M_age_* = 20.25 years, *SD* = 1.52 years), of which 387 (50.4%) were female (*M_age_* = 20.24 years, *SD* = 1.53 years).

### 3.2. Procedure

The research materials and procedures were approved by the ethics committee of Guangzhou University (protocol code: GZHU2019007; date of approval: 27 May 2019). In this study, the data were collected between 9 November and 9 December 2020. Before the formal test, the data collectors informed the participants that participation was voluntary and that they can refuse to answer questions if they feel uncomfortable. Participants were assured that their responses would be kept confidential and that they would only be used for academic research.

Mediation and moderation effects were tested with Mplus 8.3 (Muthén and Muthén, Los Angeles, CA, USA) [45]. Bootstrapping analysis with 5000 replicates was performed to verify the significance of the paths. If the confidence interval does not include 0, the path coefficient is significant. A model fit is considered acceptable when *χ*^2^/*df* is less than 5, CFI and TLI are greater than 0.90, and when RMSEA is less than 0.08, according to Hoyle’s suggestion [46]. Age, gender, family monthly income, and monthly living expenses were included in both models as control variables. We bootstrapped with 5000 samples to generate bias-corrected 95% confidence intervals. If the confidence interval excludes 0, it indicates that the parameter is statistically significant.

### 3.3. Measures

#### 3.3.1. Social Exclusion

The social exclusion questionnaire of college students was developed by Wu et al. [47]. The scale is divided into two dimensions, namely direct exclusion and indirect exclusion, and includes a total of 19 items, such as “others speak ill of me behind my back and influence other people’s views about me” (direct exclusion) and “my mistakes are coaxed or impolitely criticized” (indirect exclusion). College students were asked to report how often they experienced these situations using a 5-point Likert-type scale from 1 = very inconsistent to 5 = very consistent. The average of all items was calculated for the total score, and a higher score indicated a higher social exclusion level of college students. In this study, the scale demonstrated excellent reliability (α = 0.96).

#### 3.3.2. Self-Esteem

We used the Self-Esteem Scale compiled by Rosenberg [27] to measure self-esteem in college students. The scale includes 10 items, such as “I am able to do things as well as most other people” and “I take a positive attitude toward myself”. College students were asked to respond to items on a 4-point Likert-type scale, from 1 = highly agree to 4 = highly disagree. Reverse coding was used for some item scores and the average of all items was calculated. A higher score indicated a higher level of self-esteem in college students. In this study, the scale demonstrated excellent reliability (α = 0.86).

#### 3.3.3. Risk Preference

The 14-item risk preference questionnaire developed by Hsee and Weber [36] was used to assess the risk preference of college students, including seven profit scenario items and seven loss scenario items. Each item has two possible responses that represent a conservative choice and risk-taking choice, such as “A: 100% probability to get CNY 400, B: 50% probability to get CNY 2000, 50% probability to get CNY 0” (profit situation) and “A: 100% probability to lose CNY 600, B: 50% probability to lose CNY 2000, 50% probability to lose CNY 0” (lose situation). The probability of choosing risk option B (risk score) was used as the index of individual risk preference, that is, risk preference = the number of B/14. A higher score indicates a stronger risk preference. In this study, the scale demonstrated good reliability (α = 0.78).

#### 3.3.4. Impulsive Buying

The impulse buying intention scale was developed by Jing et al. [48] and assesses the six following dimensions: impulse buying, mood regulation, purchasing experience, consideration of the future, quick decision-making, and unplanned decision-making. There are 26 items in total, such as “I want to get what I like immediately”. College students were asked to respond to items on a 4-point Likert-type scale ranging from 1 = not at all to 7 *=* exactly. Some item scores were reversed and the average of all items was calculated. A higher average score indicated a stronger impulsive buying tendency. In this study, the scale demonstrated excellent reliability (α = 0.92).

#### 3.3.5. Control Variable

Previous studies have shown that gender, age and income are the important factors affecting impulsive buying [49,50]. In addition, because the income of Chinese college students is linked to their family monthly income and monthly living expenses, we have a certain degree of statistical control on them. In the present study, gender was dummy coded (0 = female, 1 = male). Family monthly income and monthly living expenses were both divided into four levels.

## 4. Results

### 4.1. Preliminary Analyses

We conducted a Pearson’s correlation analysis on the total average scores of social exclusion, self-esteem, impulsive buying, and risk preference. Means, standard deviations, and Pearson’s correlations (r) were calculated for all study variables in Table 1. The results show that impulsive buying was positively correlated with social exclusion (*r* = 0.36, *p* < 0.001) and risk preference (*r* = 0.12, *p* < 0.001). Self-esteem was negatively correlated with social exclusion (*r* = −0.43, *p* < 0.001) and impulsive buying (*r* = −0.22, *p* < 0.001). The means and standard deviations of the four main variables are as follows: social exclusion (mean = 1.83, *SD* = 0.65), impulsive buying (mean = 3.00, *SD* = 0.85), self-esteem (mean = 2.74, *SD* = 0.46), and risk preference (mean = 0.41, *SD* = 0.22). These findings suggest that social exclusion and low self-esteem may be predictive factors of impulsive buying and that low risk preference may be a protective factor of impulsive buying.

### 4.2. Testing for Mediation Effects of Self-Esteem

**Figure 2:**
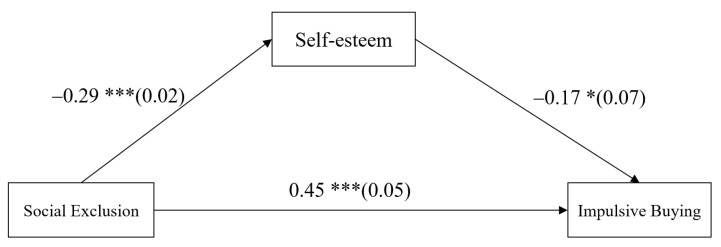
Model of the mediating role of self-esteem between social exclusion and impulsive buying. Values are unstandardized coefficients and the standard error. Paths of gender, age, family monthly income, and monthly living expenses in the model are not displayed. Of those paths, the following were significant: gender to impulsive buying (*b* = −0.20, *SE* = 0.06, *p* < 0.001, 95% CI = [−0.31, −0.10]), monthly living expenses to impulsive buying (*b* = 0.31, *SE* = 0.06, *p* < 0.001, 95% CI = [0.20, 0.42]), and family monthly income to self-esteem (*b* = 0.04, *SE* = 0.02, *p* < 0.05, 95% CI = [0.01, 0.09]). * *p* < 0.05, *** *p* < 0.001.

The mediation model represented in Figure 2 revealed an excellent fit to the data: *χ*^2^ = 3.37, *df* = 2, *χ*^2^/*df* = 1.68, CFI = 0.99, TLI = 0.98, RMSEA = 0.03. The results are displayed in Figure 2. Social exclusion negatively predicted self-esteem (*b* = −0.29, *SE* = 0.02, *p* < 0.001, 95% CI = [−0.34, −0.24]) and significantly positively predicted impulsive buying (*b* = 0.45, *SE* = 0.05, *p* < 0.001, 95% CI = [0.35, 0.56]). Self-esteem negatively predicted impulsive buying (*b* = −0.17, *SE* = 0.07, *p* < 0.05, 95% CI = [−0.31, −0.03]). Moreover, bootstrapping analyses indicated that self-esteem mediated the relationship between social exclusion and college students’ impulsive buying (indirect effect = 0.05, *SE* = 0.02, *p* < 0.05, 95% CI = [0.01, 0.09]). As covariates, gender, age, family monthly income, and monthly living expenses were included in the regression equation for control.

### 4.3. Testing for Moderated Mediation

**Figure 3:**
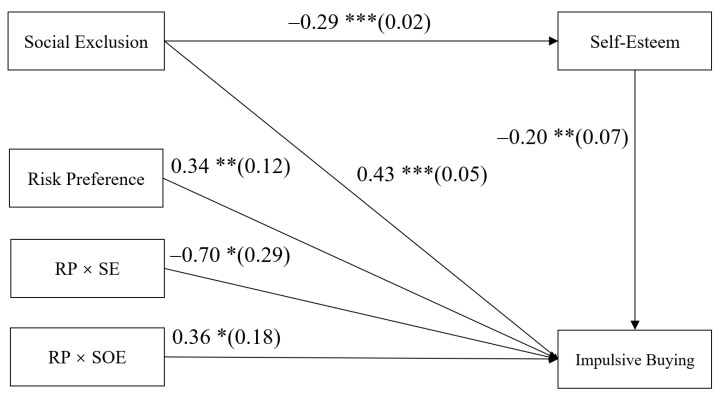
Model of the moderating role of risk preference on the direct and indirect relationship between social exclusion and impulsive buying. RP, risk preference; SE, self-esteem; SOE, social exclusion. Values are unstandardized coefficients and the standard error. Paths of gender, age, family monthly income, and monthly living expenses in the model are not displayed. Of those paths, the following were significant: gender to impulsive buying (*b* = −0.20, *SE* = 0.06, *p* < 0.001, 95% CI = [−0.30, −0.09]), and monthly living expenses to impulsive buying (*b* = 0.31, *SE* = 0.05, *p* < 0.001, 95% CI = [0.20, 0.42]). * *p* < 0.05, ** *p* < 0.01, *** *p* < 0.001.

The moderated mediation model represented in Figure 3 displayed a good fit to the data: *χ*^2^ = 36.28, *df* = 13, *χ*^2^/*df* = 2.79, CFI = 0.93, TLI = 0.91, RMSEA = 0.05. The bias-corrected percentile bootstrap results indicate that the indirect effect of social exclusion on college students’ impulsive buying through self-esteem was moderated by risk preference. Specifically, risk preference moderated the association between self-esteem and impulsive buying (*b* = −0.70, *SE* = 0.29, *p* < 0.05, 95% CI = [−1.29, −0.15]) and the association between social exclusion and impulsive buying (*b* = 0.36, *SE* = 0.18, *p* < 0.05, 95% CI = [0.00, 0.70]). As covariates, gender, age, family monthly income, and monthly living expenses were included in the regression equation for control.

**Figure 4:**
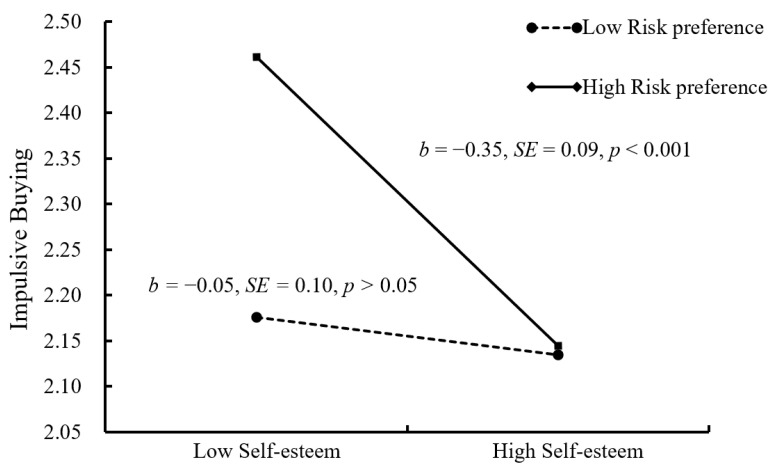
Risk preference among college students as a function of self-esteem and impulsive buying.

**Figure 5:**
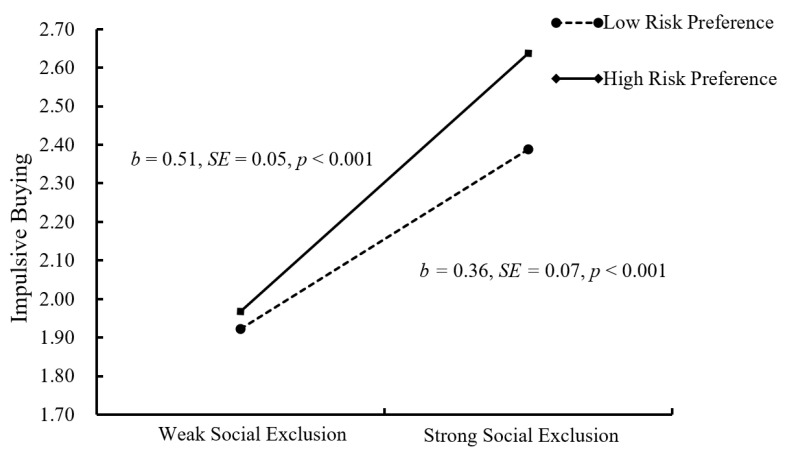
Risk preference among college students as a function of social exclusion and impulsive buying.

In order to further understand the essence of moderation, a simple slopes test was conducted in this study, and, as depicted in Figure 4 and Figure 5, the negative link between self-esteem and impulsive buying was much weaker for college students with low risk preference (1 *SD* below the mean; *b* = −0.05, *SE* = 0.10, *p* > 0.05, 95% CI = [−0.24, 0.14]) than college students with high risk preference (1 *SD* above the mean; *b* = −0.35, *SE* = 0.09, *p* < 0.001, and 95% CI = [−0.53, −0.17]). What is more, the positive link between social exclusion and impulsive buying was weaker for college students with low risk preference (1 *SD* below the mean; *b* = 0.36, *SE* = 0.07, *p* < 0.001, 95% CI = [0.22, 0.50]) than college students with high risk preference (1 *SD* above the mean; *b* = 0.51, *SE* = 0.05, *p* < 0.001, and 95% CI = [0.41, 0.61]).

Moreover, the positive indirect links between social exclusion and impulsive buying via self-esteem were much weaker for college students with low risk preference (indirect effect = 0.01, *SE* = 0.03, *p* > 0.05, 95% CI [−0.04, 0.07]) than for college students with high risk preference (indirect effect = 0.10, *SE* = 0.03, *p* < 0.001, 95% CI = [0.05, 0.16]).

## 5. Discussion

Previous empirical research has revealed there to be a relationship between social exclusion and impulsive buying. However, the internal mechanism underlying this relationship has remained unclear. Based on social exclusion theory and ecological systems theory, this study revealed the mechanism underlying the influence of social exclusion on impulsive buying. The results of this study demonstrate that social exclusion affects impulsive buying through the mediating effect of self-esteem and that the second half path and direct path of this mediating process are moderated by risk preference. The indirect and direct associations between social exclusion and impulsive buying via self-esteem were stronger among college students with high risk preference and weaker among college students with low risk preference. These research results have important theoretical significance and practical value for the prevention and intervention of impulsive buying.

### 5.1. The Relationship between Social Exclusion and Impulsive Buying

The results support the conclusion that social exclusion has a negative impact on individuals [25]. They are consistent with those of previous studies, verifying the negative impact of social exclusion on individuals as a negative factor. Previous studies have shown that social exclusion can lead to loneliness [51], depression, and anxiety [14], among other problems. College students with these problems may try to reconstruct their relationship with society by consuming luxury goods so as to enhance their sense of existence and superiority and alleviate the anxiety caused by social exclusion [52]. Combined with the results of the present study, these findings indicate that college students who are excluded from society hope to obtain instant happiness and satisfaction through impulsive buying, which makes them feel that they have reestablished contact with society. This kind of satisfaction can heighten their mood [21], which further shows that the negative impact of social exclusion has cross-domain consistency. Impulsive buying may be a compensatory mechanism for college students in the situation of social exclusion. In daily life, socially excluded college students find it more difficult to achieve a sense of existence and satisfaction. Therefore, impulsive buying makes it possible to heighten mood and gain a certain sense of value. To summarize, social exclusion is an important predictor of impulsive buying.

### 5.2. The Mediating Role of Self-Esteem

The present study found that self-esteem played a mediating role in the relationship between social exclusion and impulsive buying. Social exclusion not only directly affected impulsive buying but also indirectly affected it through self-esteem. These results indicate that social exclusion can lead to the decline of college students’ self-esteem, which will, in turn, lead to more buying behavior.

The results of this study are consistent with those of previous studies. Namely, social exclusion has been reported to affect college students’ self-esteem, reduce their sense of self-worth [14], and make them more eager to get in touch with the outside world. Adolescence is a critical period of individual psychological development, during which the influence of social relationships is crucial, even more so than that of family relationships [53]. College students in late adolescence or just after adolescence might also be more influenced by social relationships than family relationships. On the one hand, college students are eager for the acceptance and respect of peer groups; on the other hand, they can lack the ability to build strong social relationships, and so may instead gain a sense of value through impulsive and risk-taking behaviors. Social exclusion causes self-doubt and lower self-esteem in college students [14]. Compared to college students with a higher level of self-esteem, college students with lower self-esteem might seek to enhance their self-esteem; thus, they may be more likely to engage in impulsive buying behaviors. Impulsive buying can, to a certain extent, alleviate college students’ lack of a sense of value caused by lower self-esteem. Our results also show that positive self-esteem is more conducive to the ability of college students to restrain impulsive behavior, while negative self-esteem is not conducive to college students overcoming adverse reactions. Therefore, it is important to create a good social environment, which could improve the self-esteem of college students so as to avoid a series of problems. Self-esteem is of great significance to the growth and development of college students.

### 5.3. The Moderating Role of Risk Preference

This study found that risk preference played a moderating role between the direct path and the second half path of the impact of social exclusion on impulsive buying. Specifically, the direct impact of social exclusion on impulsive buying was stronger among college students with high risk preference, and the second half path of the impact of social exclusion on impulsive buying via self-esteem was also stronger among college students with high risk preference. That is, for college students with high risk preference, social exclusion had a stronger direct and indirect predictive effect on impulsive buying. Social exclusion predicted the process of impulsive buying through the mediating role of self-esteem, which was also stronger among college students with high risk preference, who are more vulnerable to low self-esteem and more social exclusion. This is consistent with previous studies, proving that lower risk preference has a certain positive effect [41].

Previous studies have shown that risk preference is a direct predictor of risk-taking and impulsive behaviors [36,37], and that risk preference has an important influence on impulsive decisions in college students. The influence of high risk preference and low risk preference on college students are also different. Namely, college students with high risk preference consider more immediate benefits brought by their decisions, including material and psychological benefits (happiness and satisfaction); college students with low risk preference pay more attention to the negative consequences of impulsive decisions which may increase their economic burden and can lead to a series of problems, such as debt [10], and so will adopt a more conservative approach when making decisions. Therefore, we believe that low risk preference, as a positive factor, can to some extent restrain college students’ impulsive behavior and the resulting series of negative consequences. College students with low self-esteem will exhibit more impulsive buying behaviors. This could be because college students with low self-esteem want to build contact with the outside world and gain a stronger identity and sense of value; thus, they tend to engage in impulsive buying behavior. As a positive factor, low risk preference can restrain these impulses, such that college students can pay more attention to the negative consequences of impulsive buying.

To summarize, it is important to educate college students about risks, especially when they make decisions that could have serious negative consequences, such as whether to engage in impulsive buying or alcohol or drug abuse. This provides a new perspective and inspiration for the development of education programs for young people. Schools and teachers should set up relevant courses to improve risk awareness in young people. We should encourage college students to not only focus on the negative consequences of impulsive behavior but also to cultivate their awareness of future prospects.

### 5.4. Limitations and Implications

While we found that social exclusion can directly predict impulsive buying, the present study has some limitations. First, although the cross-sectional design used in this study had a theoretical basis that was built on previous work and uses a self-report method, the causal relationship and internal mechanisms between variables could not be determined. Future research should choose an experimental method to test the mediating theoretical model of this study by comparing results from an experimental group and a control group. Alternatively, future research could perform longitudinal research to better test causality. Second, the subjects of this study were all college students in late adolescence or just after adolescence with small age differences, and so, the research results cannot be applied to early and middle adolescence. Future research should continue to explore the influencing mechanism underlying impulsive buying in early and middle adolescence.

Limitations aside, the findings of this study have important theoretical and practical implications. This study extends the knowledge in the impulsive buying field and contributes to our understanding of the cause of impulsive buying. Specifically, we highlighted the environmental cause (social exclusion) and cognitive cause (self-esteem) of impulsive buying, and take self-esteem into account, thus enriching the ecological model of impulsive buying. Future research should further explore the affective mechanism of college students’ impulsive buying. Moreover, the findings of this study could help to guide the targeted intervention of college students’ impulsive buying. First, the government and social organizations should strive to create a harmonious social environment and reduce the social exclusion of college students, and schools should aim to create a good school atmosphere by making certain rules [54] that help to reduce social exclusion, thereby maintaining the self-esteem of college students and reducing impulsive buying. Second, parents and education departments should cultivate students’ risk awareness, because encouraging appropriate low risk preference could help to avoid many risk behaviors in college students. Third, effective social support and psychological guidance would also help college students to make more rational shopping decisions. 

## 6. Conclusions

To summarize, this study draws the following conclusions: (1) social exclusion positively predicts college student impulsive buying; (2) self-esteem plays a mediating role in the relationship between social exclusion and impulsive buying; (3) social exclusion is moderated by risk preference through the indirect effect of self-esteem on impulsive buying, and the direct effect of social exclusion on impulsive buying is also moderated by risk preference. Specifically, in the direct path, the social exclusion of college students with high risk preference has a greater predictive effect on their impulsive buying than in college students with low risk preference. In the second half of the mediating path, the impact of social exclusion on self-esteem is stronger in college students with high risk preference, and low risk preference can significantly inhibit impulsive buying behaviors caused by low self-esteem.

## Figures and Tables

**Table 1:** Descriptive statistics and correlations for all variables.

Variables	*M*	*SD*	1	2	3	4	5	6	7	8
1. Gender	0.50	0.50	1							
2. Age	20.25	1.52	0.01	1						
3. Family monthly income	2.00	0.78	0.02	−0.01	1					
4. Monthly living expenses	1.82	0.56	−0.04	−0.05	0.43 ***	1				
5. Social exclusion	1.83	0.65	0.04	−0.07	−0.16 ***	−0.08 *	1			
6. Impulsive buying	3.00	0.85	−0.12 ***	−0.02	0.06	0.19 ***	0.36 ***	1		
7. Self-esteem	2.74	0.46	0.02	0.07 *	0.15 ***	0.08 *	−0.43 ***	−0.22 ***	1	
8. Risk preference	0.41	0.22	0.03	−0.03	0.04	0.07	0.04	0.12 ***	−0.03	1

Note: Gender was dummy-coded: 1 = male, 0 = female; * *p* < 0.05, *** *p* < 0.001.

## Data Availability

The data presented in this study are available upon request from the corresponding author.
